# In Vitro Modulation of Redox and Metabolism Interplay at the Brain Vascular Endothelium: Genomic and Proteomic Profiles of Sulforaphane Activity

**DOI:** 10.1038/s41598-018-31137-7

**Published:** 2018-08-23

**Authors:** Ravi K. Sajja, Mohammad A. Kaisar, Vikrant Vijay, Varsha G. Desai, Shikha Prasad, Luca Cucullo

**Affiliations:** 1 Department of Pharmaceutical Sciences, Texas Tech University Health Sciences Center, Amarillo, TX 79106 USA; 2 Department of Neurology, Northwestern University - The Feinberg School of Medicine, Chicago, IL 60611 USA; 3 Division of Systems Biology, National Center for Toxicological Research, US FDA, Jefferson, AR 72079 USA; 4 Center for Blood Brain Barrier Research, Texas Tech University Health Sciences Center, Amarillo, TX 79106 USA

## Abstract

Sulforaphane (SFN) has been shown to protect the brain vascular system and effectively reduce ischemic injuries and cognitive deficits. Given the robust cerebrovascular protection afforded by SFN, the objective of this study was to profile these effects *in vitro* using primary mouse brain microvascular endothelial cells and focusing on cellular redox, metabolism and detoxification functions. We used a mouse MitoChip array developed and validated at the FDA National Center for Toxicological Research (NCTR) to profile a host of genes encoded by nuclear and mt-DNA following SFN treatment (0–5 µM). Corresponding protein expression levels were assessed (ad hoc) by qRT-PCR, immunoblots and immunocytochemistry (ICC). Gene ontology clustering revealed that SFN treatment (24 h) significantly up-regulated ~50 key genes (>1.5 fold, adjusted p < 0.0001) and repressed 20 genes (<0.7 fold, adjusted p < 0.0001) belonging to oxidative stress, phase 1 & 2 drug metabolism enzymes (glutathione system), iron transporters, glycolysis, oxidative phosphorylation (OXPHOS), amino acid metabolism, lipid metabolism and mitochondrial biogenesis. Our results show that SFN stimulated the production of ATP by promoting the expression and activity of glucose transporter-1, and glycolysis. In addition, SFN upregulated anti-oxidative stress responses, redox signaling and phase 2 drug metabolism/detoxification functions, thus elucidating further the previously observed neurovascular protective effects of this compound.

## Introduction

Constituted by the specialized brain microvascular endothelial phenotype, the blood-brain barrier (BBB) protects and preserves the central nervous system (CNS) sanctuary^1–3^. To maintain its dynamic and metabolic functions, the BBB endothelium is abundantly enriched with mitochondria and glucose transporter (such us glucose transporter 1 - Glut1 ~55 kD) (25; 80) which sustain the endothelial bioenergetic activity. These mitochondria also play a critical role in redox metabolism and detoxification functions. Furthermore, the BBB endothelium displays abundant expression of various solute carrier family and ATP-dependent transporters, besides tight junction assemblies, for enhanced nutrient/solute uptake, utilization and transport to the brain^4–6^. Strong causative link between oxidative and pro-inflammatory stresses, brain microvascular dysfunction, and CNS disease^7^ Overarching evidence indicates that sustained oxidative stress generated by pro-oxidant and pro-inflammatory stimuli, can negatively impact BBB physiology and function^3,8–11^.

In this respect, sulforaphane, a naturally occurring organosulfur compound generated from cruciferous vegetables (e.g., broccoli), is a potent inducer of antioxidant^12^ and phase-2 detoxifying enzymes^13^. As such, this compound has received a great deal of attention for its neuroprotective effects both *in vitro* and *in vivo*^14–18^. Consistent with its antioxidative and protective activities to counteract oxidative damage inflammatory challenges, several studies have clearly shown that SFN affords cytoprotection largely through nuclear factor-erythroid 2-related factor 2 (Nrf2)-dependent mechanisms^14,17,19–21^. In fact, SFN is a well-known pharmacologic promoter/activator of Nrf2^18,22^. Nrf2 is a ubiquitously expressed redox-sensitive transcription factor that acts as a master regulator of the antioxidative response system implicated in redox homeostasis, anti-inflammatory activity, detoxification and radical scavenging functions as well as cellular bioenergetics^6,23,24^. By disrupting Keap1 activity, SFN blocks polyubiquitination and consequent degradation of Nrf2 thus allowing for Nrf2 to translocate and accumulate in the nucleus and initiate its transcriptional program^25^. More recently we demonstrated the neurovascular/BBB protective role of Nrf2^2,18^, whereas induction of Nrf2 by SFN has been clearly shown to promote BBB integrity and afford neuroprotection against ischemic injuries^15,18^.

Interestingly recent reports have also outlined the concomitantly to activation of the cellular antioxidative defenses^20,26^, SFN can modulate mitochondrial dynamics^27,28^. This is a crucial yet under-investigated aspect of SFN activity. In fact, mitochondria generate more than 90% of the energy by oxidative phosphorylation for the cell. These organelles are also intricately involved in other key metabolic pathways, such as fatty acid oxidation, the Krebs cycle, the urea cycle, heme biosynthesis, iron and calcium homeostasis, and steroid biosynthesis^29^. While mitochondrial function is vital for cell survival these organelles play a significant role in the execution of apoptosis (programmed cell death), a process for the removal of potentially dangerous cells^30^ as well as cellular detoxification. Collectively, these functions highlight a critical role of mitochondria in the life and death of the cell. Considering their major role in energy production, tissues such as brain, liver, heart, respiratory systems, skeletal muscle, kidney, and endocrine glands significantly depend on mitochondria for their high energy demands. Therefore, dysfunction in these organelles could potentially lead to various degenerative diseases and disorders in these organs as well as various drug-induced toxicities, as several therapeutic drugs are known to target mitochondria^31,32^. Based on these well-established evidences, we sought to profile the impact of SFN on nuclear and mitochondrial DNA gene expression and to investigate SFN modulation of cellular redox and metabolism interplay at the brain vascular endothelium.

A distinctive feature of mitochondria is that they contain their own DNA that encodes 13 important proteins essential for energy production while the rest of approximately 78 proteins are encoded by the nuclear DNA. Additionally, more than 1500 proteins essential for various metabolic pathways within mitochondria are encoded by nuclear DNA. This suggests that the precise coordination between the mitochondrial and the nuclear genomes is crucial to meet the energy demands of the cell or tissues as well as for efficient functioning of mitochondria. Because of the complexity of mitochondria, the exact role of mitochondria in development of number of degenerative diseases or drug toxicities is still not well understood. To address this knowledge gap, a comprehensive transcriptomics tool, MitoChip was designed and developed that consists of genes encoded by both genomes associated with various mitochondrial pathway/biological processes including, oxidative phosphorylation, fatty acid β-oxidation, Krebs cycle, heme biosynthesis, steroid biosynthesis, mitochondrial biogenesis, mitochondrial fusion and fission, mitochondrial DNA transcription, replication and repair, apoptosis, and various mitochondrial membrane transporters^33^. Unlike commercial high-density whole genome expression arrays, MitoChip contains genes encoded by both genomes to understand a cross-talk between two genomes during various disease conditions or toxic insults. In this study we focused our attention on a specific subset of targets encompassing enzymes function in the redox metabolism, antioxidative response and detoxification systems. For example glutathione S-transferase alpha (**GSTAs**) and glutathione S-transferase mu types (**GSTMs**) are phase 2 enzymes involved with xenobiotic metabolism and oxidative stress. Glutathione S-transferase pi 1 (**GSTP1**) is also a phase 2 enzyme which catalyzes the conjugation of hydrophobic and electrophilic compounds with reduced glutathione. Catalase (**CAT**) and Thioredoxin 1 (**Txn1** are key enzymes involved with redox biology whereas CAT promotes the decomposition of hydrogen peroxide to water and oxygen, thus protecting the cell from oxidative damage by ROS. Hexokinase Type I (**Hk1**) and pyruvate dehydrogenase kinase 1 (**Pdk1**) are enzymes responsible for the regulation of homeostasis of carbohydrate fuels and glycolysis/energy homeostasis, etc. In addition, we look at a major regulator of cerebral iron homeostasis such as ferroportin (**Slc40a1**) also implicated in the modulation of mitochondrial redox metabolism and cell death^34^.

For these purposes ue used a mouse MitoChip array encompassing a total of 1,027 unique genes associated with mitochondrial structure and function^35,36^ to obtain important insights into the effects of SFN. In parallel, by western blotting, real-time quantitative reverse transcription polymerase chain reaction (qRT-PCR) and integrated cell culture (ICC), we investigated the cellular impact of SFN treatment using mouse primary brain microvascular endothelial cells.

## Results

### SFN affects the expression level of brain vascular endothelial mitochondria-related genes and Nrf2/Nqo1 upregulation

**Figure 1:**
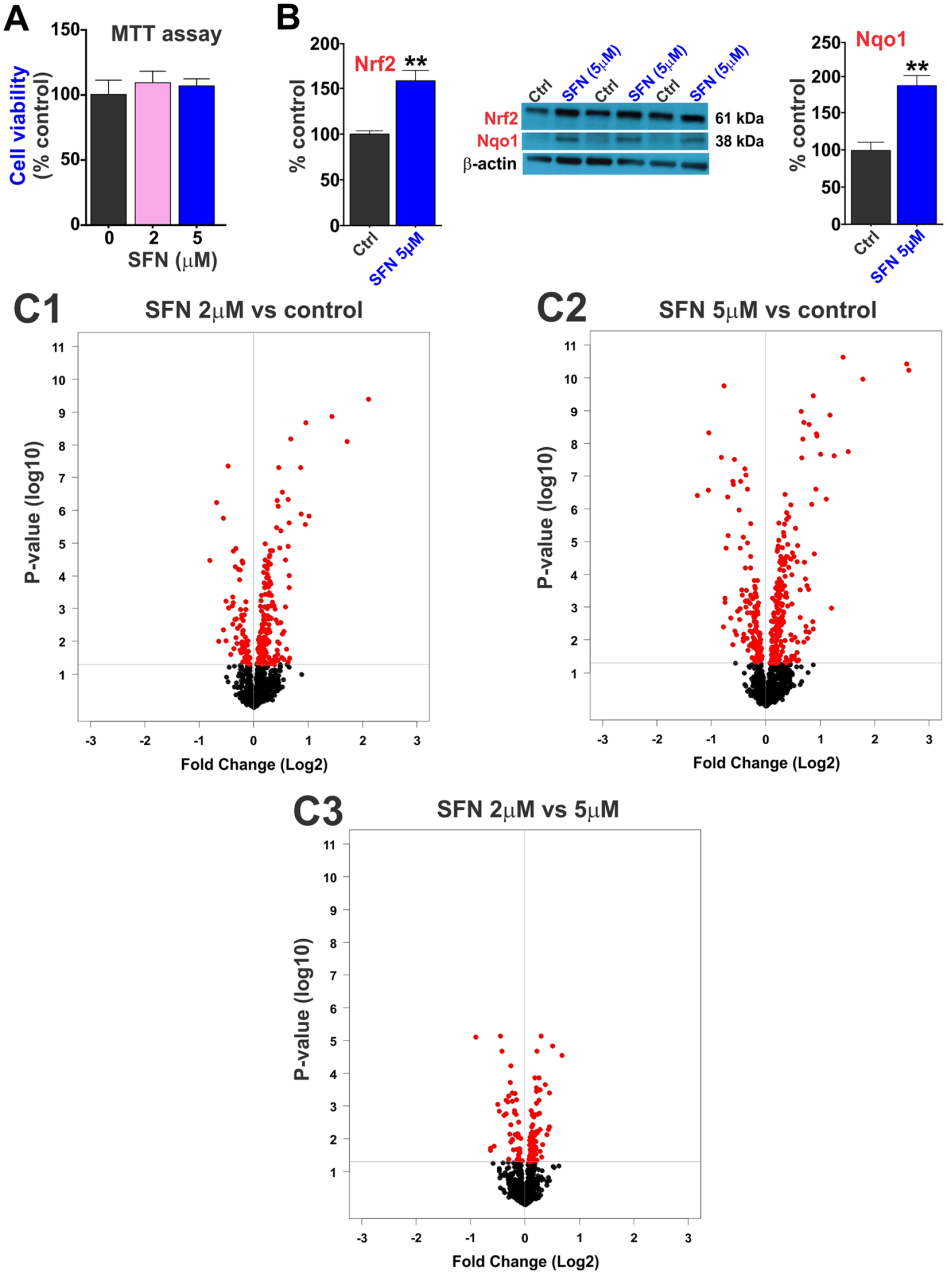
SFN impact on mitochondria-related gene expression. (**A1**) Cell viability was not affected by SFN (5 µM) as determined by MTT assay. (**A2**) SFN treatment upregulates the expression level of Nrf2 and its downstream target Nqo1 in mice brain microvascular endothelial cells. (**B1**–**B3**) Volcano plots of gene expression in response to different concentration of SFN (**B1**) 2 µM vs. control; (**B2**) 5 µM vs control and (**B3**) 2 vs. 5 µM) as analyzed by MitoChip microarray in primary cells. Horizontal line represents p = 0.05. Genes with expression not significantly altered are indicated by solid black circles and genes with expression significantly altered are indicated by solid red circles. Significantly down-regulated genes are in the upper left quadrant and significantly up-regulated genes are in the upper right quadrant. N = 3–4 independent biological samples per condition repeated in triplicates. Blots of Nrf2, Nqo1 and β-actin were taken from different gels.

As demonstrated by the 3-(4,5-dimethylthiazol-2yl)-2,5-diphenyltetrazolium bromide (**MTT**) assay (Fig. 1A), SFN does not impact cell viability at any of the concentrations tested and significantly upregulates Nrf2 expression and its downstream target NAD(P)H: Quinone oxidoreductase 1 (**Nqo1**; Fig. 1B). The relative impact of SFN on mitochondria-specific gene expression is evident when looking at the Volcano plots (Fig. 1C1–C3). These Volcano plots represent the effect of SFN on the expression levels of several of mitochondria-specific genes (see also Supplementary Table 2). Fold changes are plotted on X-axis while significance or p-values are plotted on the Y-axis. If we compare the position and density of the red dots, it’s clear that the effect is dose-dependent with increases in the expression levels and significance of the changes (see also Supplementary Table 1). A total of 50 genes were upregulated by more than 1.5-fold whereas around 20 genes were repressed down to less than 0.7-fold. Mitochondria-specific genes are highly conservative compared to other nuclear genes^37^ and a change in 1.5-fold is considered as highly significant.

### SFN promotes ATP production through upregulation of key enzymes involved in glucose transport and utilization via glycolytic pathway

**Figure 2:**
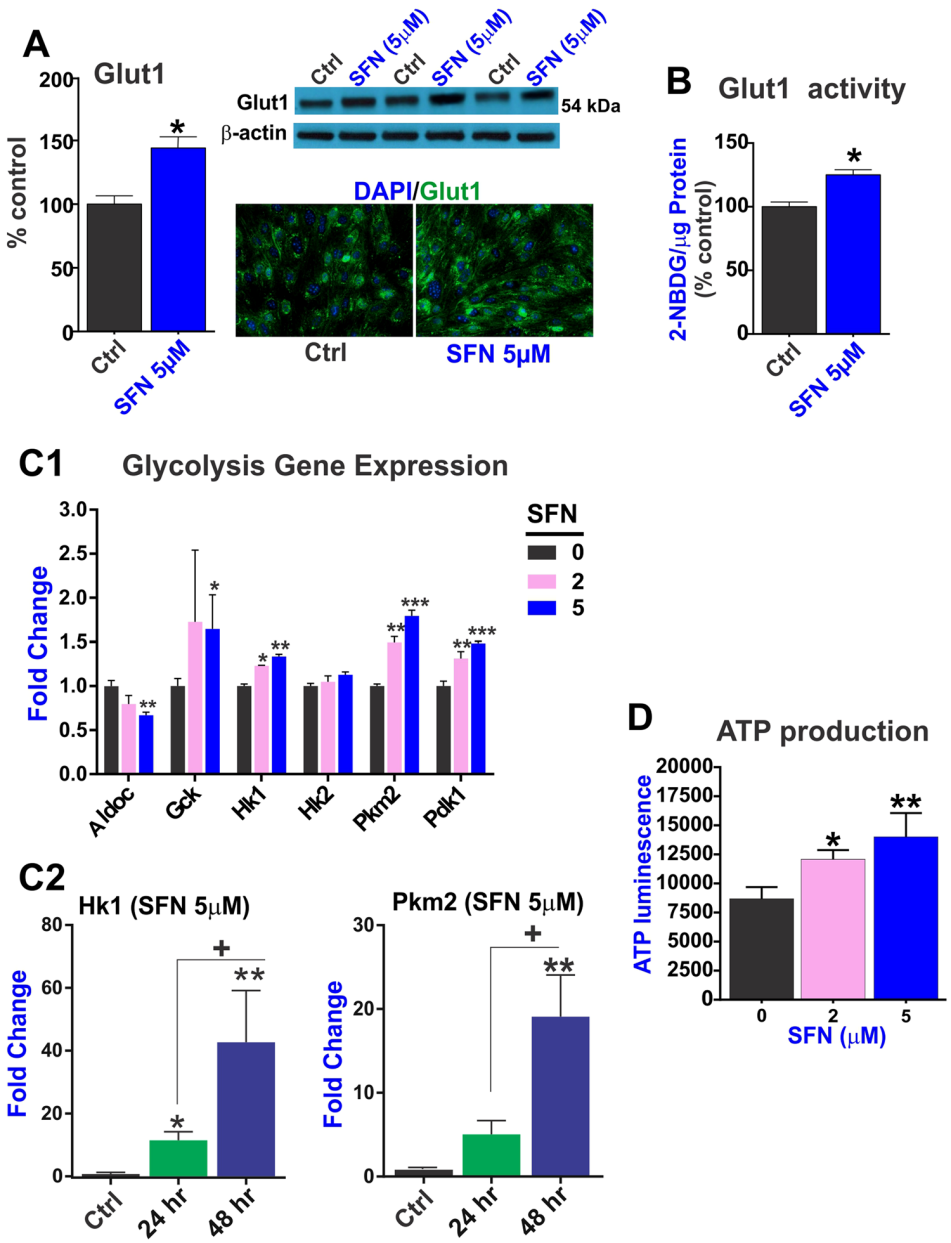
SFN modulate the redox-metabolic interplay of the brain vascular endothelium to promote ATP production. SFN promotes upregulation of Glut1 expression (**A**) and transport activity (**B**) as determined by 2-NBDG uptake normalized to total protein. (**C**) Key genes involved in the regulation of glycolysis were also upregulated including hexokinase 1 (**Ht1**) and pyruvate kinase 2 (**Pkm2**) as analyzed by MitoChip array (**C1**) and confirmed by RT-PCR (**C2**). The overall cellular effect was a substantial increase in ATP production (**D**). N = 3 independent biological samples per condition assayed in triplicates. “*”p < 0.05, “**”p < 0.01 compared to controls (fold changes). For the time-dependent study (24 vs. 48 h SFN treatment) “+” p < 0.05 vs. 24 h. Blots of Glut1 and β-actin were taken from different gels.

To maintain its dynamic and metabolic functions, the brain microvascular endothelium at the BBB is abundantly enriched with glucose transporter 1 (**Glut1**) protein (~55kD)^38^. Here, we show that SFN promotes Glut1 expression and activity (Fig. 2) in brain vascular endothelium. 24 h exposure to SFN (5 µM) promoted the expression of the glucose transporter Glut1 as demonstrated by western blots (WB) analyses and immunofluorescence in mouse BBB endothelial cells (Fig. 2A). The effect was not limited to Glut1 expression levels but also extended to its overall transport activity as determined by 2-(N-(7-Nitrobenz-2-oxa-1,3-diazol-4-yl)Amino)-2-Deoxyglucose (**2-NBDG**) uptake (p < 0.05 vs. control; see Fig. 2B).

Following influx into the cytoplasm, glucose undergoes glycolysis to ultimately produce ATP. Hexokinases are the first members of this cycle. Pdk1 plays a key role in regulation of glucose and fatty acid metabolism and homeostasis via phosphorylation of the pyruvate dehydrogenase subunits PDHA1 and PDHA2 while **Hk1** is one of four hexokinase isoenzymes that participate in glycolysis and catalyzes the phosphorylation of glucose into glucose-6-phosphate (G6P)^39^ to initiate cellular respiration, thus providing enough activation energy to jumpstart the glycolytic process. As shown in Fig. 2C1,C2, SFN significantly upregulated the gene expression level of Hk1 as compared to Hk2 (Fig. 2C1) which was further validated by RT-PCR analysis (Fig. 2C2). Similarly, the gene expression of Pdk1 (a mitochondrial multienzyme complex that catalyzes the oxidative decarboxylation of pyruvate) one of the major enzymes responsible for the regulation of homeostasis of carbohydrate fuels was upregulate by SFN.

Mitochip analysis also showed that the level of glucokinase (**Gsk**; another hexokinase with a high Km for glucose) was also upregulated by SFN (although results were statistically significant only at the high concentration of 5 µM). The role of GCK is to provide G6P for the synthesis of glycogen.

Pyruvate kinase isozyme type 2 (**Pkm2**) is a rate-limiting enzyme that catalyzes the last step of the glycolysis cascade and is responsible for net ATP production^40^. Similarly, SFN treatment upregulated Pkm2 gene expression (Fig. 2C1) and results were also verified by RT-PCR analysis (Fig. 2C2). In this respect it is important to note that significant difference in the magnitude of change in gene expression measured by qPCR and MitoChip microarray is attributed to the difference in the dynamic range of detection between the two techniques (like any DNA microarray, the MitoChip is a transcriptomics tool that allows simultaneous measurement of expression of thousand mitochondria-related genes; however, with a smaller dynamic range compared to qPCR).

As shown in Fig. 2D, the overall impact of SFN on cellular bioenergetics was indeed a significant dose-dependent increase in ATP production which was observed at 48 hours exposure (24 hours SFN exposure did not elicit statistically significant changes – data not shown). This finding further corroborates previous data linking SFN with mitochondrial respiration and bioenergetics^41^.

### SFN promotes redox signaling and the expression of phase 2 drug metabolism enzymes

**Figure 3:**
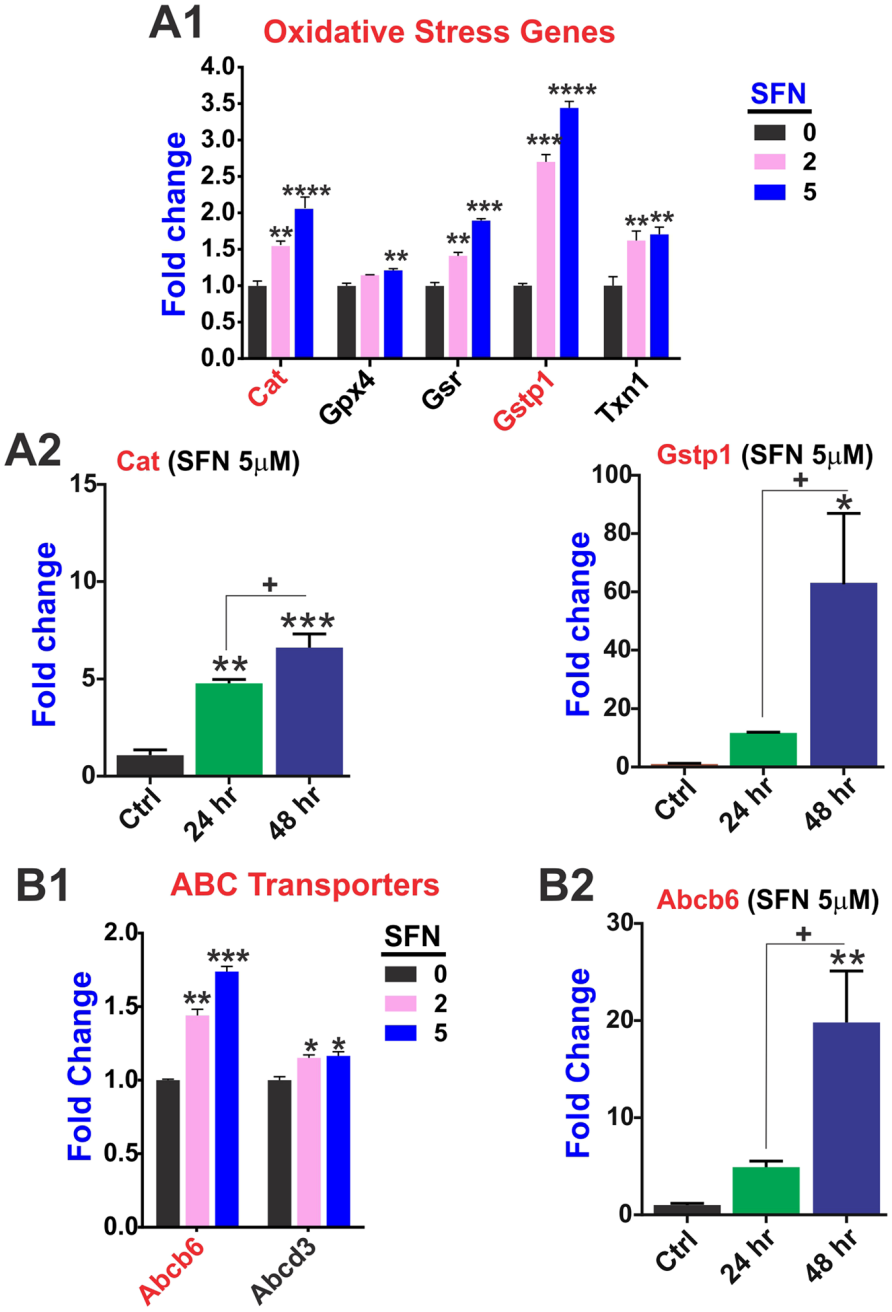
SFN promotes the expression of genes involved in oxidative stress responses and redox signaling. MitoCHIP array-based analysis of anti-oxidative gene expression changes (**A1**). Most prominent/relevant gene expression were validated ad hoc by RT-PCR (**A2**). SFN treatment up-regulated the expression of Abc transporters (**B1**) and most relevant gene expression changes were validated ad hoc by RT-PCR (**B2**). N = 4 biological replicates per condition (assayed in triplicates). *p < 0.05, **p < 0.005 and ***p < 0.001 vs. controls (fold changes).

Our MitoChip data clearly shows that SFN significantly upregulated (in a dose-dependent manner) the expression level of catalase (**Cat**) along with that of several glutathione S-transferases (**GSTs**) family members including Gstp1 (a phase 2 enzyme involved in xenobiotic metabolism), glutathione-disulfide reductase (**GSR**), and thioredoxin 1 (**Txn1**) (Fig. 3A1). Cat is a key antioxidant enzyme responsible for the conversion of hydrogen peroxide to water and oxygen and thereby mitigates the toxic effects of this highly reactive oxidant substance. Complementary to Cat’s activity, **GST1** is a major detoxifying enzyme that catalyzes the conjugation of endogenous and exogenous toxicants with reduced glutathione. Impact of SFN on the gene expression data of these 2 major detoxification enzymes were further validated ad hoc by RT-PCR analysis (Fig. 3A2).

Results from the mouse MitoChip gene expression arrays also revealed a significant SFN dose-dependent up-regulation of Abc transporters Abcd3 and particularly Abcb6 (Fig. 3B1). The effect in the latter case was both dose and time-dependent and resulted in a nearly 20-fold increase in Abcb6 expression (vs. control) at 48 h exposure (Fig. 3B2). Data were analyzed by one-way ANOVA followed by Tukey’s *post hoc* test.

### SFN upregulates the iron transporter Slc40a1 and the cellular oxidative stress response system

**Figure 4:**
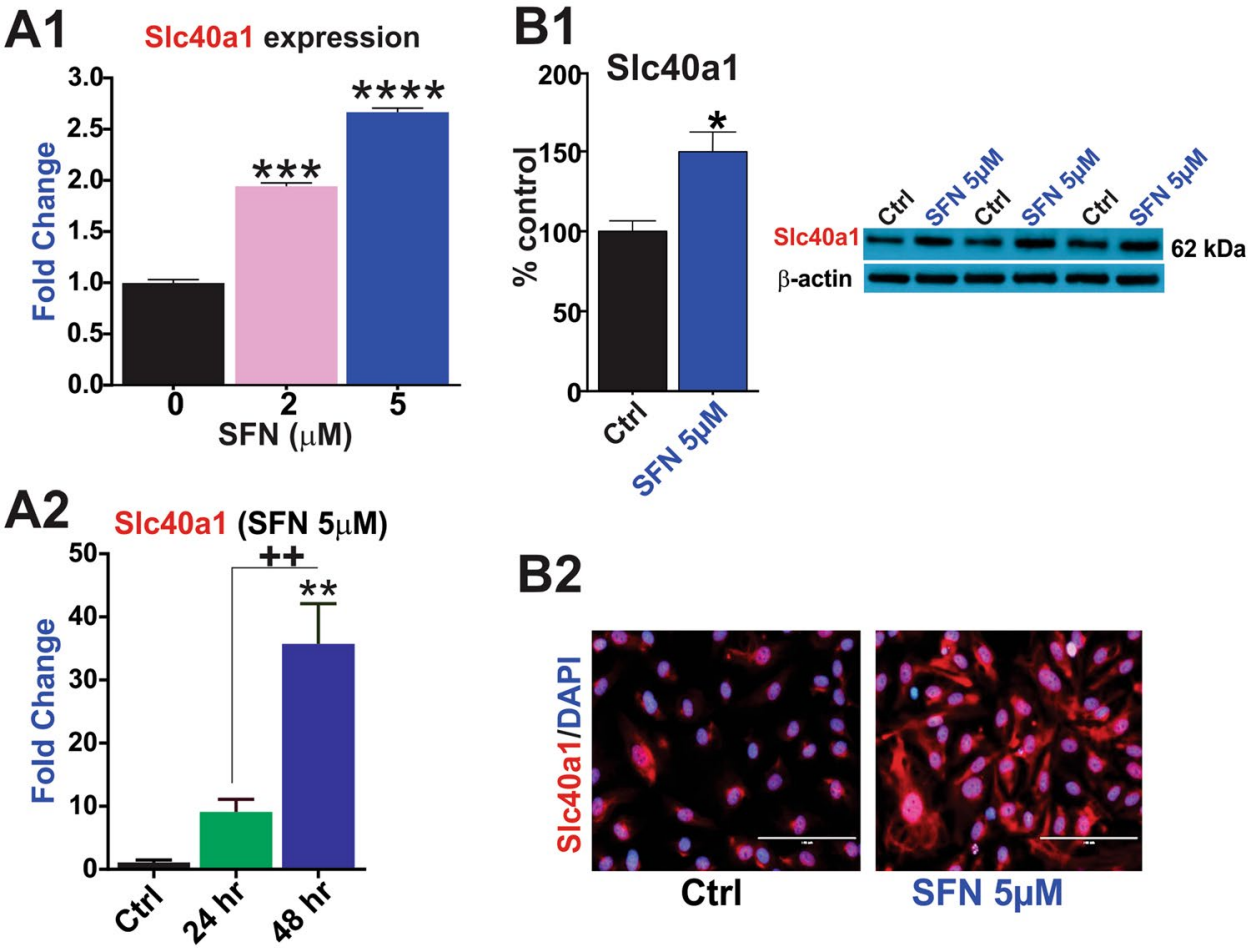
SFN promotes the expression of the Slc40a1 iron importer in brain vascular endothelial cells. SFN promotes the transcription of the Slc40a1 iron exporter gene (fold change) as shown by MitoChip array (**A1**) and RT-PCR analysis (**A2**). Results were further confirmed through measurements of Slc40a1 protein expression by western blots (**B1**) and immunofluorescence (**B2**). Image scale = 100 µm at 40x magnification. N = 3–4 biological replicates per condition (assayed in triplicates). **p < 0.01, ***p < 0.001 and ****p < 0.0001, vs. controls. Blots of Slc40a1 and β-actin were taken from different gels.

Our mouse MitoChip assay revealed dose- and time-dependent regulation of several Slc transporters by SFN (see also Supplementary Table 3). Notably, SFN upregulated the expression of Slc40a1 (solute carrier family 40 member 1 or better known as ferroportin-1; see Fig. 4A1; p < 0.005 and p < 0.001 vs. control). Slc40a1 upregulation was both dose- and time-dependent (Fig. 4A2) with the most significant changes observed following 48 hours of SFN treatment. Experiments were further validated by WB (Fig. 4B1) and immunofluorescence (Fig. 4B2) analyses.

**Figure 5:**
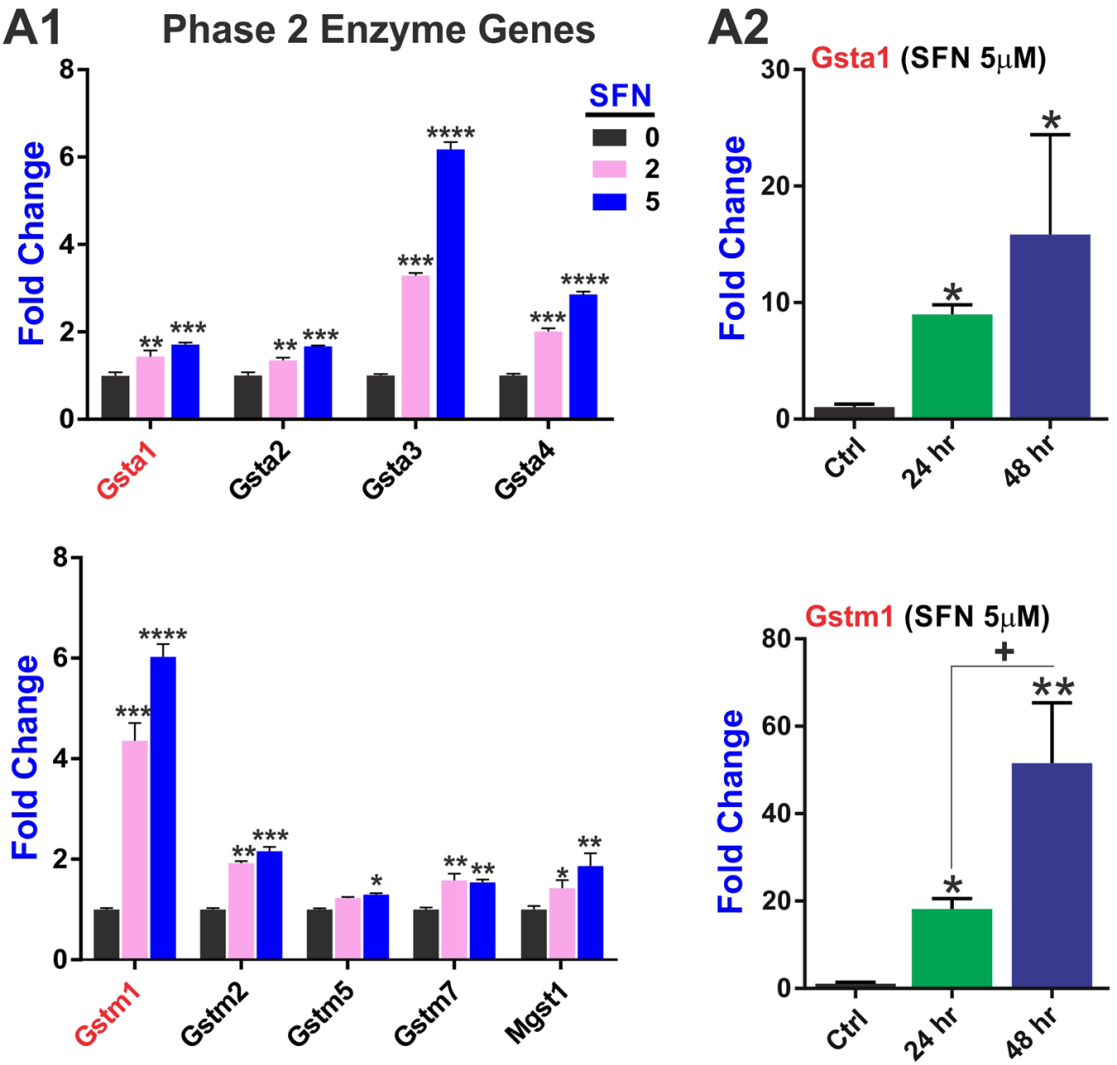
SFN regulates the expression of phase 2 drug metabolism/detoxification genes of the glutathione system in a dose-dependent manner. (**A1**) MitoChip array data of GSTs enzymes involved in cellular detoxification that were affected by SFN. Changes in the gene expression levels of relevant GST enzymes (Gsta1 and Gstm1) were validated ad hoc by RT-PCR (**A2**). Results demonstrated a dose and time-dependent effect of SFN treatment over upregulation of these enzymes. N = 4 samples per condition and assayed in triplicates. *p < 0.05, **p < 0.005 and ***p < 0.001 compared to controls (fold changes).

In addition, we looked at the SFN impact on phase 2 drug metabolism/detoxification genes of the glutathione system. Our results clearly (Fig. 5A1) indicate that SFN effectively promotes the upregulation of a class of phase 2 enzyme glutathione S-Transferases of the alpha (Gsta1–4) and mu (Gstm1, 2, 5, 7) types which function in the detoxification of electrophilic compounds. In addition, the expression level of the microsomal glutathione S-transferase 1 (**Mgst1**; also involved in cellular defense against toxic, carcinogenic, and pharmacologically active electrophilic compounds) was upregulated. Mitochip array data were verified (ad hoc) by RT-PCR (Fig. 5A2) demonstrating that the observed effects were both dose- and time-dependent.

## Discussion

Our data demonstrates that SFN can positively influence the redox-metabolic interplay at the brain microvascular endothelium (via Nrf2 modulation) and effectively promote glycolysis and ATP production to help sustaining the highly energy demanding functions of these cells.

Our results show that SFN promotes a cascade of bioenergetic events that ultimately leads to increased production of ATP (see also Fig. 2D) and cytoprotection from oxidative stress. This is evident when we look at these effects in the proper contest starting with: (a) upregulation of Glut 1 expression and activity (Fig. 2A and B) which facilitate glucose entry followed by upregulation of Hk1 (Fig. 2C) which converts glucose into glucose 6 phosphate, thus allowing to maintain a positive gradient for glucose. Hk1 also exhibits a pivotal role in maintaining the homeostasis of mitochondria by restraining ROS generation and conserving mitochondrial membrane potential (**Δψ**_**m**_)^39^. Glucose entry is then complemented by upregulation of Pkm2, a limiting glycolytic enzyme that catalyzes the final step in glycolysis (Fig. 2C) resulting in an increased production of ATP (Fig. 2D) which was evident following 48 h SFN exposure but not at 24 h (data not shown). This is likely due to the fact that ATP synthesis is the end term of the metabolic process and a significant increase of ATP must be preceded by an amplification of the various bioenergetic steps involved in its production and thus takes longer to become manifest. Concurrently, the metabolic processes involved in the synthesis of ATP also provide extra reductive equivalent (in the form of NADPH) and facilitate the generation of reduced glutathione to counteract cellular ROS and oxidative stress^42,43^.

### SFN positively impacts redox metabolism

Cytoprotective functions include control of redox metabolism and detoxification systems which encompass efflux transporters and phase 2 drug metabolism enzymes. Our data have clearly shown that SFN promotes the expression levels of several major antioxidative stress and detoxification enzymes including Gstp1, Cat, Gsr, and Txn1 (see Fig. 4). Specifically, Gstp1 is a phase 2 enzyme which catalyzes the conjugation of hydrophobic and electrophilic compounds with reduced glutathione. Loss of Gstp1 has been associated with nuclear oxidative stress damage and potentiation of cell injury induced by ROS^44,45^. Cat promotes the decomposition of hydrogen peroxide to water and oxygen, thus protecting the cell from oxidative damage by ROS^46,47^. Glutathione-disulfide reductase (**GSR**) instead catalyzes the reduction of glutathione disulfide (**GSSG**) to its sulfhydryl and active form GSH which is necessary to maintain the reducing environment of the cells and resist oxidative stress^48^. Finally, Txn1 is a small redox protein which has been shown to exert neuroprotection effects against cerebral ischemia/reperfusion injury caused by oxidative stress^49^ and is also crucial to NK cells to resist H_2_O_2_-induced cell death^50^. Thus, SFN-mediated upregulation of Gstp1 Cat, Gsr, and Txn1 among other antioxidative enzymes clearly show the potential protective potential of this substance.

Among the ABC family of transporters (Fig. 3B1,B2) SFN is clearly able to promote upregulation Abcb6, a porphyrin transporter (importer) which plays a major role in cell survival responses^51,52^ and cellular resistance to metal-induced cellular toxicity^53,54^. In addition to Abcb6, SFN also promoted the expression levels of Abcd3 (albeit to a lesser extent) (Fig. 4B1). This is also of interest since not only Abcd3 has a prominent role in cellular oxidative stress^55,56^ but also has a tangible presence in brain cells including glial cells. In fact recent studies showed that knockdown of Abcd3 in glial cells markedly elevate oxidative stress responses^57^ and its expression was also significantly down-regulated in a cellular model of ischemic/reperfusion injury studied in brain endothelial cells^55^. As a matter of fact SFN has been shown to protect the neurovascular system in stroke^18^ as well as neurons exposed to oxygen and glucose deprivation such us during an ischemic injury^16^. In light of these findings, further studies specifically focused on Abcd3 and its transcriptional regulation by SFN would be critical to understanding its role in brain cytoprotection and injuries by oxidative stress.

In addition to Abc transporters, SFN also upregulated Slc40a1 (also known as Ferroportin 1; a well-studied iron importer) which plays a critical role in cerebral iron homeostasis and has been implicated with several neurological disorders including Alzheimer’s disease brain pathology^58–60^, abnormal forebrain patterning and neural tube closure and inflammation^61,62^. This is of relevant importance given the inference of iron overload in cellular damage^34^ and further outlines the protective potential of SFN.

### SFN upregulates the detoxification systems

Another facet of SFN activity encompasses phase 2 detoxification systems and specifically GSTs enzymes. These are a group of glutathione S-transferases expressed in different subcellular compartments including cytosol and mitochondria^63,64^ and are involved in oxidative stress responses, detoxification functions but also cell signaling and disease prevention/progression. Our results clearly show that SFN induced the upregulation of several of GST enzymes involved in the detoxification and defense against reactive compounds including xenobiotics and endogenous metabolic bioproducts^65^ (Fig. 5; see also Supplementary Table 4). This is of importance given their intimate relation to neurological and neurodegenerative disorders including Parkinson disease and epilepsy^66,67^. The specific GST isoforms most affected by SFN include the glutathione S-Transferases of the alpha (Gsta1–4) and mu (Gstm1, 2, 5, 7) types which are involved in the detoxification of active electrophilic compounds by conjugation with glutathione. GST isoenzyme Gstm1 has also been shown to protect cells against TGF beta- and ischemia/reperfusion-induced apoptosis^68^ and its antioxidative activity has been recently associated with lower HIV disease progression and severity in HIV infected patients^69^.

In addition to GSTs enzymes, noteworthy is the fact that SFN also increased the gene expression level of the microsomal glutathione S-transferase 1 (**Mgst1**; Fig. 5A1 bottom panel) Mgst1 is a homotrimeric protein with glutathione transferase and peroxidase activities^70^ particularly important in the central nervous system in relation to age-related increase of ROS which plays a major role as prodromal factor for the onset of neurodegenerative disorders^71^. In fact, SFN has been shown to reactivate the antioxidant response system during aging^26^.

### Conclusion and final remarks

Oxidants and electrophiles arising from internal metabolism and xenobiotic sources play an important role in maintaining physiological functions, cell signaling, and cellular defense mechanisms. In conclusion, excessive generation of ROS by both internal and external factors initiates events such as anti-oxidant depletion, lipid peroxidation, and cellular toxicity thereby creating a state of redox imbalance^72^. From a cerebrovascular and CNS perspective, a growing body of evidence indicate that oxidants and electrophiles are among the principal mediators in the initiation and progression of several cerebrovascular and neurodegenerative diseases. The brain microvascular endothelium at the BBB displays abundant expression of various solute carrier family and ATP-dependent transporters, besides tight junction assemblies, for enhanced nutrient/solute uptake, utilization and transport to the brain^38^.

In this contest, activation of the antioxidative response system by SFN can provide an effective counteractive tool against oxidative stress and afford protection against systemic inflammatory challenges, reperfusion/cerebrovascular injuries, and neurodegenerative diseases^14,16–21,73^. On these lines, stimulation of redox activities and cellular metabolism by SFN helps supporting the high demand for bioenergetic fuel (in the form of ATP) and redox cytoprotection from ROS and highly reactive electrophilic substances. Thus, SFN can effectively sustain critical cellular counteractive responses to xenobiotics, oxidative stress stimuli while promoting cell viability (see Sup. Fig. 6). This latter also includes regulation of mitochondrial dynamics (encompassing fusion, fission and biogenesis) and mitochondrial transcription factors (see Sup. Fig. 7) to support the cellular activities.

While Nrf2-dipendent and independent activity of SFN have been previously demonstrated^73^, we did not investigate the specific impact of these modulatory pathways since it was deemed outside the scope of this work. However, we recognize that this is an important aspect of SFN activity that will need to be addressed in future studies. In addition, *in vivo* studies will be necessary to assess the broader impact of the observed changes in mitochondrial functions and cellular response across the entire neurovascular unit.

## Methods

### Materials and reagents

L-Sulforaphane (SFN) was purchased from Sigma-Aldrich (St. Louis, MO). Rabbit anti-Nrf2 (#sc-722) and mouse anti-Nqo1 (#sc-376023) antibodies were from Santa Cruz Biotechnology (Dallas, TX); rabbit anti-Slc40a1 was from Novus Biologicals (Littleton, CO). Mouse anti-Glut1 (clone 5B12.3) from EMD Millipore (Billerica, MA); Mini-Protean® TGXTM gels for western blotting were purchased from Bio-rad (Hercules, CA, USA). Donkey anti-rabbit (#NA934) and sheep anti-mouse (#NA931) secondary antibodies were from GE Healthcare (Piscataway, NJ); Alexa Fluor^®^ 488 or 555 conjugated goat anti-rabbit or anti-mouse antibodies were obtained from Invitrogen (Carlsbad, CA). Sterile culture wares were purchased from Fisher Scientific (Pittsburgh, PA, USA) while molecular biology grade reagents and chemicals were purchased from Sigma-Aldrich (St. Louis, MO, USA) or Bio-rad laboratories (Hercules, CA, USA).

### Cell Culture

Mouse primary brain microvascular endothelial cells (mBMECs, #C57-6023) from Cell Biologics (Chicago, IL, USA) were expanded (p3-6) in complete endothelial growth medium (Cell Biologics).

### Drug treatment

Following an overnight incubation in treatment media with reduced serum, cells were treated with 0–5 µM SFN for 24 h^73^.

### Cell Viability

The effects of SFN treatment (24 h) on cell viability (metabolic activity) were determined by MTT (3-(4,5-dimethylthiazol-2yl)-2,5-diphenyltetrazolium bromide) assay^74^ and CellTiter-Glo® 2.0 ATP luminescence assay (Promega), using BioTek Synergy 2 microplate reader^75^.

### Immunofluorescence

Following drug treatment, cells were rinsed with PBS and fixed with ice-cold 4% buffered formalin for 10–15 min. Cells were then washed 3X with PBS. Later cells were permeabilized using 0.02% triton 100X and subsequently blocked with 10% goat serum in PBS followed by overnight incubation at 4 °C with primary antibodies in blocking buffer (1:100–150) reactive against human or mouse proteins (Glut1, Nrf2, Nqo1 and Slc40a1). Next day, cells were washed with blocking buffer and incubated for 1 h with Alexa Fluor 488 or 555-conjugated anti-rabbit or anti-mouse antibodies (1:1000) at room temperature. Cells were washed with PBS, dried at room temperature and cover-slipped with DAPI in Prolonged Gold Anti-fade reagent (Invitrogen, Carlsbad, CA). Images were captured using an EVOS XL digital inverted fluorescence microscope at 40 × (Advanced Microscopy Group - AMG, Cat #AME3301).

### Western Blotting

Total protein was collected by cell lysis in RIPA buffer with protease inhibitors and quantitated by BCA assay. Equal amounts of protein across samples were subjected to SDS-PAGE separation (4–20% graded gels) as described earlier^76^. Following gel to PVDF membrane electrotransfer of the protein bands, membranes were blocked with blocking buffer (0.1% tween-20 in TBS) containing 5% non-fat dry milk and 1% BSA. Subsequently, membranes were incubated overnight at 4 °C with primary antibodies in blocking buffer (in range of 1:200-1-500), washed repeatedly and probed with HRP-conjugated secondary antibodies (1:5000). Bands were visualized by chemiluminescence reagent using X-ray film-based autoradiography and densities were analyzed by Li-Cor Image Studio software with β-actin as a loading control.

### Glucose uptake assay

To assess the BBB endothelial glucose transporter (Glut1) activity, we adapted a previously reported procedure for glucose uptake with minor modifications^77^. Briefly, following treatment, cells were washed with DPBS buffer and incubated in warm transport buffer (10 mM HEPES and 0.2% BSA in EBM2) containing 2-NBDG ((2-(N-(7-Nitrobenz-2-oxa-1,3-diazol-4-yl)Amino)-2-Deoxyglucose; Molecular Probes, #N13195) at 10 µM for 15 min. Cells were repeatedly washed with ice-cold HBSS and lysed with RIPA buffer for 10 min. NBD fluorescence was determined in the lysates using BioTek Synergy 2 microplate reader (Ex/Em = 470/540 nm) and normalized against protein content.

### Mouse MitoChip gene expression array

To assess the transcriptional role of Nrf2 in the regulation of diverse gene networks specific to mitochondrial structure and function in the BBB endothelium (mitochondriome), we employed a custom-designed mouse MitoChip arrays (Agilent Technologies Inc., Santa Clara, CA) developed by National Center for Toxicological Research^33^. It consists of both gDNA and mtDNA encoding 812 genes associated with mitochondrial structure and function, 70 genes involved in both intrinsic and extrinsic apoptotic pathways, 117 genes involved in phase I (cytochrome P450 oxidoreductases), phase II (glutathione S-transferases, UDP-glucuronidases, sulfuryl transferases) of drug metabolism, and 20 housekeeping genes to give a total of 1,019 unique genes on the MitoChip. This is the upgrade of the previous mouse MitoChip of 542 mitochondria-related genes developed for evaluation of mitochondrial role in various drug-induced organ toxicities^35,36^. Following treatment, total RNA was isolated from primary cells by RNAeasy plus mini kit (Qiagen Inc, Germantown, MD) with genomic DNA decontamination^78^. RNA integrity and yield was determined by Nanodrop™ ND-1000 (Thermo Fisher Scientific, Waltham, MA) and Agilent 2100 Bioanalyzer (Agilent Technologies Inc.). cDNA strand synthesis, labeling, and hybridization were performed at the Nationwide Children’s Hospital (Columbus, OH) in a random design of samples on mouse MitoChip. A complete gene list of Mitochip genes is provided in Supplementary Table 4. Following hybridization, arrays were scanned using the Agilent DNA Microarray Scanner (Agilent Technologies Inc., Santa Clara, CA 95051, US). The images were analyzed using Agilent’s Feature Extraction software (V10.7.3). Significant changes in the expression of important genes following normalization as observed on MitoChip with an absolute fold change of 1.25 and false discovery rate < 0.05 were further validated by quantitative real-time PCR^35^.

### Quantitative RT-PCR

Total RNA (1 µg) extracted as mentioned above was reverse transcribed to cDNA by Superscript III first strand synthesis kit (Life Technologies, Carlsbad, CA). The cDNA strands were mixed with gene-specific forward and reverse primer pairs (Integrative DNA technologies, Coralville, IA; Table 1) and SYBR select master mix (Life Technologies). Template-free and RT-negative samples served as internal controls. Amplification was performed in triplicates/sample on Bio-Rad CFX96 Touch Real-Time PCR system (95 °C for 5 min followed by 40 cycles of 95 °C-30 sec, 58 °C-1 min and 72 °C-1 min and a terminal reaction at 72 °C-2 min). The threshold values (counts, Ct) for target genes and reference genes Rpl21) were determined for each sample and relative expression of each target gene was calculated by ^ΔΔ^Ct method.

**Table 1:** Forward and reverse (5′-3′) primer sequences used for qRT-PCR experiments.

Mouse gene	Forward	Reverse
Gstp1	TGTAATCGGCAAAGGAGATCTG	TCACCCTCATCTACACCAACT
Abcb6	GCCGTGATATGAACACACAG	GCCAAAAAGCACAAAGTCCC
Catalase	AAATGCTTCAGGGCCGCCTT	GTAGGGACAGTTCACAGGTA
Hk1	CGTCAAGATGCTGCCAACCT	GCACGATGTTCTCTGGGGTG
Nrf2	TCAAACACTTCTCGACTTACTCC	TGATGGACTTGGAGTTGCC
Gstm1	AGGTGTTGCGATGTAGCG	CCTGCTTATGACATTCTTGACC
Gsta1	TGCCCAATCATTTCAGTCAG	CCAGAGCCATTCTCAACTA
Rpl21	CCATAAGTGCTACCACGGCA	GCCCTTCTCTTTGGCTTCCT
Slc40a1	TGTCAGCCTGCTGTTTGCAGGA	TCTTGCAGCAACTGTGTCACCG
Pgc1b	TCCTGTAAAAGCCCGGAGTAT	GCTCTGGTAGGGGCAGTGA
Pkm2	TCGCATGCAGCACCTGATT	CCTCGAATAGCTGCAAGTGGTA
Nqo1	CGCCTGAGCCCAGATATTGT	GGAAAGGACCGTTGTCGTAC

### Data Analysis

Results are reported as mean ± SEM and analyzed for statistical significance by one-way ANOVA followed by Dunnett’s or Tukey’s multiple comparison tests using GraphPad Prism Software Inc. (La Jolla, CA, USA). Student’s t-test (two-tailed, unpaired) was used to compare control vs. treatment effects. P value < 0.05 was considered for statistical significance.

#### MitoChip gene expression

Data extraction, pre-processing, normalization, and statistical comparison to obtain differential gene expression were performed using SAS 9.4 (SAS Institute Inc., Cary, NC). The intensity (gProcessedSignal) data for all samples were extracted from raw data files (generated by Agilent Feature Extraction software). The intensity data for all 1019 genes from all arrays were transformed to log2 values. The mean intensity of six replicates for each gene was calculated and used as the intensity for a respective gene. Then, an array-wise 50th percentile normalization was performed. Generalized linear model procedure (PROC GLM) in SAS was used to measure statistical significance (p < 0.05) of the contrast between control and different SFN treatment groups to determine differentially expressed genes. A relative fold change in expression level of each gene was calculated as a difference in average log2 intensity values of two comparison groups. Also, raw p-values were adjusted to False Discovery Rate (FDR). An absolute fold change greater than 1.25 and FDR < 5% was used as criteria for differentially expressed genes.

#### Gene Ontology analysis of MitoChip data

The interpretation of large amounts of complex microarray gene expression data and translating it into biologically meaningful information can be challenging. However, Gene Ontology (GO) has increasingly been used to find patterns within groups of genes^79^. The GO is organized as a hierarchy of annotation terms that facilitate the analysis and interpretation at different levels such as molecular function, cellular compartment, and biological process. It is a powerful tool that enables one to classify genes according to mined functional categories and statistically analyze the obtained classifications, leading to a better understanding and interpretation of microarray data.

Mitochondria-related GO terms based on biological processes or molecular functions were thus obtained from the Mouse Genome Informatics (www.informatics.jax.org) database and NCBI GO annotation (ftp://ftp.ncbi.nlm.nih.gov/gene/DATA/gene2go.gz) database. The overall significance of treatment effect on each GO term was measured by a modified meta-analysis method to combine p-values calculated for each gene within the GO term while considering the inter-gene correlation structure in each GO term^80^.

## Electronic supplementary material

Supplementary information

Dataset

## Data Availability

The datasets generated and/or analyzed during the current study are available from the corresponding author on reasonable request.

## References

[1] Festoff BW, Sajja RK, van Dreden P, Cucullo L (2016). HMGB1 and thrombin mediate the blood-brain barrier dysfunction acting as biomarkers of neuroinflammation and progression to neurodegeneration in Alzheimer’s disease. J Neuroinflammation.

[2] Prasad S (2017). Role of Nrf2 and protective effects of Metformin against tobacco smoke-induced cerebrovascular toxicity. Redox Biol.

[3] Sajja RK, Rahman S, Cucullo L (2016). Drugs of abuse and blood-brain barrier endothelial dysfunction: A focus on the role of oxidative stress. J Cereb Blood Flow Metab.

[4] Helms HC (2016). *In vitro* models of the blood-brain barrier: An overview of commonly used brain endothelial cell culture models and guidelines for their use. J Cereb Blood Flow Metab.

[5] Miller DS (2015). Regulation of ABC transporters blood-brain barrier: the good, the bad, and the ugly. Adv Cancer Res.

[6] Wang X (2014). Nrf2 upregulates ATP binding cassette transporter expression and activity at the blood-brain and blood-spinal cord barriers. J Neurosci.

[7] Zlokovic BV (2008). The blood-brain barrier in health and chronic neurodegenerative disorders. Neuron.

[8] Kebir H (2007). Human TH17 lymphocytes promote blood-brain barrier disruption and central nervous system inflammation. Nat Med.

[9] Rochfort KD, Collins LE, Murphy RP, Cummins PM (2014). Downregulation of blood-brain barrier phenotype by proinflammatory cytokines involves NADPH oxidase-dependent ROS generation: consequences for interendothelial adherens and tight junctions. Plos One.

[10] Rochfort KD, Cummins PM (2015). The blood-brain barrier endothelium: a target for pro-inflammatory cytokines. Biochem Soc Trans.

[11] Rochfort KD, Cummins PM (2015). Cytokine-mediated dysregulation of zonula occludens-1 properties in human brain microvascular endothelium. Microvasc Res.

[12] Kraft AD, Johnson DA, Johnson JA (2004). Nuclear factor E2-related factor 2-dependent antioxidant response element activation by tert-butylhydroquinone and sulforaphane occurring preferentially in astrocytes conditions neurons against oxidative insult. J Neurosci.

[13] Thimmulappa RK (2002). Identification of Nrf2-regulated genes induced by the chemopreventive agent sulforaphane by oligonucleotide microarray. Cancer Res.

[14] Tarozzi A (2013). Sulforaphane as a potential protective phytochemical against neurodegenerative diseases. Oxid Med Cell Longev.

[15] Mao, L. *et al*. Protective effects of sulforaphane in experimental vascular cognitive impairment: Contribution of the Nrf2 pathway. *J Cereb Blood Flow Metab*, 271678X18764083, 10.1177/0271678X18764083 (2018). 10.1177/0271678X18764083 PMC6365596 29533123

[16] Soane L, Li Dai W, Fiskum G, Bambrick LL (2010). Sulforaphane protects immature hippocampal neurons against death caused by exposure to hemin or to oxygen and glucose deprivation. J Neurosci Res.

[17] Yu C (2017). Sulforaphane improves outcomes and slows cerebral ischemic/reperfusion injury via inhibition of NLRP3 inflammasome activation in rats. Int Immunopharmacol.

[18] Alfieri A (2013). Sulforaphane preconditioning of the Nrf2/HO-1 defense pathway protects the cerebral vasculature against blood-brain barrier disruption and neurological deficits in stroke. Free Radic Biol Med.

[19] Yamagishi S, Matsui T (2016). Protective role of sulphoraphane against vascular complications in diabetes. Pharm Biol.

[20] Matsui T, Nakamura N, Ojima A, Nishino Y, Yamagishi SI (2016). Sulforaphane reduces advanced glycation end products (AGEs)-induced inflammation in endothelial cells and rat aorta. Nutr Metab Cardiovasc Dis.

[21] Zhao X, Wen L, Dong M, Lu X (2016). Sulforaphane activates the cerebral vascular Nrf2-ARE pathway and suppresses inflammation to attenuate cerebral vasospasm in rat with subarachnoid hemorrhage. Brain Res.

[22] Dinkova-Kostova AT, Kostov RV (2012). Glucosinolates and isothiocyanates in health and disease. Trends Mol Med.

[23] Holmstrom KM (2013). Nrf2 impacts cellular bioenergetics by controlling substrate availability for mitochondrial respiration. Biol Open.

[24] Hayes JD, Dinkova-Kostova AT (2014). The Nrf2 regulatory network provides an interface between redox and intermediary metabolism. Trends Biochem Sci.

[25] Kensler TW (2013). Keap1-nrf2 signaling: a target for cancer prevention by sulforaphane. Top Curr Chem.

[26] Kubo E, Chhunchha B, Singh P, Sasaki H, Singh DP (2017). Sulforaphane reactivates cellular antioxidant defense by inducing Nrf2/ARE/Prdx6 activity during aging and oxidative stress. Sci Rep.

[27] de Oliveira, M. R., de Bittencourt Brasil, F. & Furstenau, C. R. Sulforaphane Promotes Mitochondrial Protection in SH-SY5Y Cells Exposed to Hydrogen Peroxide by an Nrf2-Dependent Mechanism. *Mol Neurobiol*, 10.1007/s12035-017-0684-2 (2017). 10.1007/s12035-017-0684-2 28730528

[28] O’Mealey GB, Berry WL, Plafker SM (2017). Sulforaphane is a Nrf2-independent inhibitor of mitochondrial fission. Redox Biol.

[29] Schatz G (1995). Mitochondria: beyond oxidative phosphorylation. Biochim Biophys Acta.

[30] Petit P. X. K. G. In *Mitochondrial DNA Mutations in Aging*, *Disease and Cancer* (ed Singh, K. K.) 147–165 (Springer, 1998).

[31] Nadanaciva S, Will Y (2011). New insights in drug-induced mitochondrial toxicity. Curr Pharm Des.

[32] Wallace KB (2015). Multiple Targets for Drug-Induced Mitochondrial Toxicity. Curr Med Chem.

[33] Vijay V (2016). Early transcriptional changes in cardiac mitochondria during chronic doxorubicin exposure and mitigation by dexrazoxane in mice. Toxicol Appl Pharmacol.

[34] Bogdan AR, Miyazawa M, Hashimoto K, Tsuji Y (2016). Regulators of Iron Homeostasis: New Players in Metabolism, Cell Death, and Disease. Trends Biochem Sci.

[35] Desai VG, Fuscoe JC (2007). Transcriptional profiling for understanding the basis of mitochondrial involvement in disease and toxicity using the mitochondria-specific MitoChip. Mutat Res.

[36] Desai VG (2007). Development of mitochondria-specific mouse oligonucleotide microarray and validation of data by real-time PCR. Mitochondrion.

[37] Wolstenholme DR (1992). Animal mitochondrial DNA: structure and evolution. Int Rev Cytol.

[38] Abbott NJ, Patabendige AA, Dolman DE, Yusof SR, Begley DJ (2010). Structure and function of the blood-brain barrier. Neurobiol Dis.

[39] da-Silva WS (2004). Mitochondrial bound hexokinase activity as a preventive antioxidant defense: steady-state ADP formation as a regulatory mechanism of membrane potential and reactive oxygen species generation in mitochondria. J Biol Chem.

[40] Dong G (2016). PKM2 and cancer: The function of PKM2 beyond glycolysis. Oncol Lett.

[41] Carrasco-Pozo C, Tan KN, Gotteland M, Borges K (2017). Sulforaphane Protects against High Cholesterol-Induced Mitochondrial Bioenergetics Impairments, Inflammation, and Oxidative Stress and Preserves Pancreatic beta-Cells Function. Oxid Med Cell Longev.

[42] Anastasiou D (2011). Inhibition of pyruvate kinase M2 by reactive oxygen species contributes to cellular antioxidant responses. Science.

[43] Brimacombe, K. R. *et al*. In *Probe Reports from the NIH Molecular Libraries Program* (2010). 21433368

[44] Fletcher ME (2015). Influence of glutathione-S-transferase (GST) inhibition on lung epithelial cell injury: role of oxidative stress and metabolism. Am J Physiol Lung Cell Mol Physiol.

[45] Mian OY (2016). GSTP1 Loss results in accumulation of oxidative DNA base damage and promotes prostate cancer cell survival following exposure to protracted oxidative stress. Prostate.

[46] Ciriolo MR, Marasco MR, Iannone M, Nistico G, Rotilio G (1997). Decrease of immunoreactive catalase protein in specific areas of ageing rat brain. Neurosci Lett.

[47] Giordano CR (2015). Catalase therapy corrects oxidative stress-induced pathophysiology in incipient diabetic retinopathy. Invest Ophthalmol Vis Sci.

[48] Deponte M (2013). Glutathione catalysis and the reaction mechanisms of glutathione-dependent enzymes. Biochim Biophys Acta.

[49] Wang M, Zhu K, Zhang L, Li L, Zhao J (2016). Thioredoxin 1 protects astrocytes from oxidative stress by maintaining peroxiredoxin activity. Mol Med Rep.

[50] Mimura K (2017). Upregulation of thioredoxin-1 in activated human NK cells confers increased tolerance to oxidative stress. Cancer Immunol Immunother.

[51] Boswell-Casteel RC, Fukuda Y, Schuetz JD (2017). ABCB6, an ABC Transporter Impacting Drug Response and Disease. AAPS J.

[52] Lynch J, Fukuda Y, Krishnamurthy P, Du G, Schuetz JD (2009). Cell survival under stress is enhanced by a mitochondrial ATP-binding cassette transporter that regulates hemoproteins. Cancer Res.

[53] Cordova EJ (2014). The NRF2-KEAP1 pathway is an early responsive gene network in arsenic exposed lymphoblastoid cells. Plos One.

[54] Zhang YK (2017). Establishment and characterization of arsenic trioxide resistant KB/ATO cells. Acta Pharm Sin B.

[55] Lindner C (2012). ATP-binding cassette transporters in immortalised human brain microvascular endothelial cells in normal and hypoxic conditions. Exp Transl Stroke Med.

[56] Porcellotti S (2015). Oxidative Stress during the Progression of beta-Amyloid Pathology in the Neocortex of the Tg2576 Mouse Model of Alzheimer’s Disease. Oxid Med Cell Longev.

[57] Di Benedetto R, Denti MA, Salvati S, Attorri L, Di Biase A (2009). PMP70 knock-down generates oxidative stress and pro-inflammatory cytokine production in C6 glial cells. Neurochem Int.

[58] Crespo AC (2014). Genetic and biochemical markers in patients with Alzheimer’s disease support a concerted systemic iron homeostasis dysregulation. Neurobiol Aging.

[59] Brown DE (2015). Increased ferroportin-1 expression and rapid splenic iron loss occur with anemia caused by Salmonella enterica Serovar Typhimurium infection in mice. Infect Immun.

[60] Raha AA, Vaishnav RA, Friedland RP, Bomford A, Raha-Chowdhury R (2013). The systemic iron-regulatory proteins hepcidin and ferroportin are reduced in the brain in Alzheimer’s disease. Acta Neuropathol Commun.

[61] Urrutia P (2013). Inflammation alters the expression of DMT1, FPN1 and hepcidin, and it causes iron accumulation in central nervous system cells. J Neurochem.

[62] Mao J (2010). The iron exporter ferroportin 1 is essential for development of the mouse embryo, forebrain patterning and neural tube closure. Development.

[63] Raza H, Robin MA, Fang JK, Avadhani NG (2002). Multiple isoforms of mitochondrial glutathione S-transferases and their differential induction under oxidative stress. Biochem J.

[64] Raza H (2011). Dual localization of glutathione S-transferase in the cytosol and mitochondria: implications in oxidative stress, toxicity and disease. FEBS J.

[65] Hayes JD, Flanagan JU, Jowsey IR (2005). Glutathione transferases. Annu Rev Pharmacol Toxicol.

[66] Shang W (2008). Expressions of glutathione S-transferase alpha, mu, and pi in brains of medically intractable epileptic patients. BMC Neurosci.

[67] Shi M (2009). Identification of glutathione S-transferase pi as a protein involved in Parkinson disease progression. Am J Pathol.

[68] Yuan XP (2017). MicroRNA-423-5p facilitates hypoxia/reoxygenation-induced apoptosis in renal proximal tubular epithelial cells by targeting GSTM1 via endoplasmic reticulum stress. Oncotarget.

[69] Parsons, M. *et al*. Effect of GSTM1-Polymorphism on Disease Progression and Oxidative Stress in HIV Infection: Modulation by HIV/HCV Co-Infection and Alcohol Consumption. *J AIDS Clin Res* **4**, 10.4172/2155-6113.1000237 (2013). 10.4172/2155-6113.1000237 PMC3887471 24416632

[70] Johansson K, Jarvliden J, Gogvadze V, Morgenstern R (2010). Multiple roles of microsomal glutathione transferase 1 in cellular protection: a mechanistic study. Free Radic Biol Med.

[71] von Bernhardi R, Tichauer JE, Eugenin J (2010). Aging-dependent changes of microglial cells and their relevance for neurodegenerative disorders. J Neurochem.

[72] Ma Q, He X (2012). Molecular basis of electrophilic and oxidative defense: promises and perils of Nrf2. Pharmacol Rev.

[73] Holloway PM (2016). Sulforaphane induces neurovascular protection against a systemic inflammatory challenge via both Nrf2-dependent and independent pathways. Vascul Pharmacol.

[74] Naik P (2014). Oxidative and pro-inflammatory impact of regular and denicotinized cigarettes on blood brain barrier endothelial cells: is smoking reduced or nicotine-free products really safe?. BMC Neurosci.

[75] Prasad S, Sajja RK, Kaisar MA, Cucullo L (2016). Hyperglycemia exacerbates antiretroviral drug combination induced blood-brain barrier endothelial toxicity. Neurotoxicology.

[76] Prasad S (2015). Impact of cigarette smoke extract and hyperglycemic conditions on blood-brain barrier endothelial cells. Fluids Barriers CNS.

[77] Abdul Muneer PM, Alikunju S, Szlachetka AM, Murrin LC, Haorah J (2011). Impairment of brain endothelial glucose transporter by methamphetamine causes blood-brain barrier dysfunction. Mol Neurodegener.

[78] Sajja RK, Green KN, Cucullo L (2015). Altered Nrf2 signaling mediates hypoglycemia-induced blood-brain barrier endothelial dysfunction *in vitro*. Plos One.

[79] Beissbarth T (2006). Interpreting experimental results using gene ontologies. Methods Enzymol.

[80] Lee T, Desai VG, Velasco C, Reis RJ, Delongchamp RR (2008). Testing for treatment effects on gene ontology. BMC Bioinformatics.

