# Supplementary material for: In Vitro Modulation of Redox and Metabolism Interplay at the Brain Vascular Endothelium: Genomic and Proteomic Profiles of Sulforaphane Activity
Source: Sci Rep. 2018 Aug 23;8:12708. doi: 10.1038/s41598-018-31137-7 (PMC6107504; doi:10.1038/s41598-018-31137-7)
Supplement: Supplementary file 1 — Supplementary information [file 41598_2018_31137_MOESM1_ESM.docx]

**In Vitro Modulation of Redox and Metabolism Interplay at the Brain Vascular Endothelium: Genomic and Proteomic Profile of Sulforaphane Activity**

Ravi K. Sajja1, Mohammad A. Kaisar1, Vikrant Vijay3, Varsha G. Desai3, Shikha Prasad2 , Luca Cucullo1,4*****

**Supplementary Material**

**
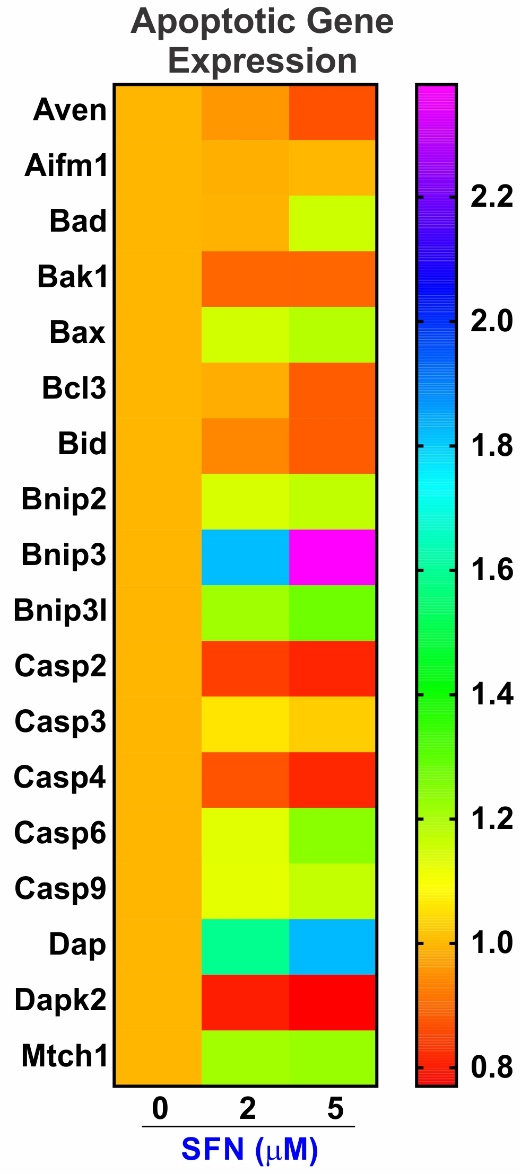
**

**Supplementary Figure 6:** SFN regulation of apoptotic genes (**B**).

**
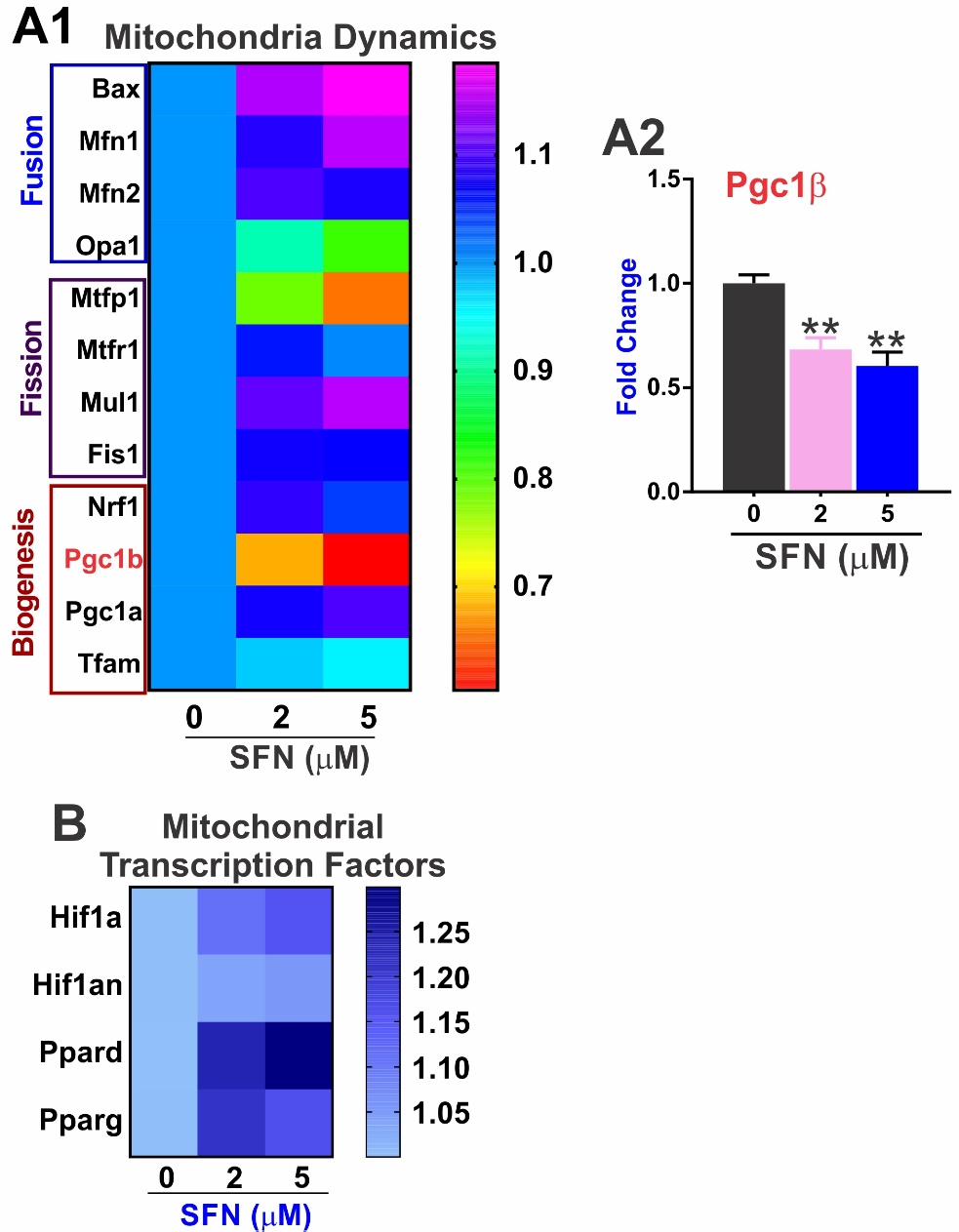
**

**Supplementary Figure 7:** **SFN impacts mitochondrial dynamics.** **A1)** Analysis of the heatmap shows how SFN differentially impact the expression levels of genes involved in mitochondrial fission, fusion and biogenesis. **A2)** Representative RT-PCR bar graph showing then effect of SFN treatment on Pgc1β expression. **B)** Heatmap of SFN regulation of mitochondrial transcriptional factors. Data were obtained from two independent experiments (n= 4 biological replicates/each). “******” p<0.01 vs. control.

**Supplementary-Table 2: Excel List of all the 1026 genes included on MitoChips.** The attached excel spreadsheet contains to separate sheets: The first one “**LS Means per gene per group**” contains the “mean” gene expression data for each experimental group including gene name, gene description and whether is encoded in the nucleus or the mitochondria. The second one “**MitoChip data comparison**” contains the data relative to the experimental comparisons shown in the volcano plots. The following is the column legend for the above mention excel file.

| **GENEBANKACC** | Contains Genbank Accession or Refseq Gene IDs |
| --- | --- |
| **GENENAME** | Contains Entrez Gene Symbols |
| **GEN_DESCR_MFR** | Contains expanded Gene Name |
| **MitoEncoded** | "**1**" represents that the gene is encoded by mitochondria and "**0**" represents nuclear-encoded genes |
| **LOCUSID** | Nuclear-encoded genes |
| **fold_ratio_linear** | Contains Entrez Gene IDs |
| **raw_p** | Fold change |

**Supplementary-Table 3:** Effects of SFN on gene expression of mitochondrial transporter/carrier systems

| **Gene Bank Acc** | **Gene** | **SFN2/ctrl** | | **SFN5/ctrl** | |
| --- | --- | --- | --- | --- | --- |
|  |  | **Abs FC** | **Fdr_P** | **Abs FC** | **Fdr_P** |
| NM_053196 | Sideroflexin 2 (sfxn2) | -1.61 | <0.001 | -2.08 | <0.0001 |
| NM_007472 | Aquaporin 1 (Aqp1) | 1.56 | 0.002 | 1.63 | <0.0001 |
| NM_019687 | Solute carrier family 22 (organic cation transporter), member 4 (Slc22a4) | -1.43 | 0.009 | -1.39 | 0.007 |
| NM_153150 | Solute carrier family 25, member 1 (Slc25a1) | 1.19 | 0.07 | 1.24 | 0.015 |
| NM_013770 | Solute carrier family 25 (mitochondrial carrier; dicarboxylate transporter), member 10 (Slc25a10) | -1.1 | 0.08 | -1.26 | 0.048 |
| NM_175194 | Solute carrier family 25 (mitochondrial carrier; Graves’ disease autoantigen), member 16 (Slc25a16) | -1.16 | 0.06 | -1.25 | 0.005 |
| NM_030054 | Solute carrier family 25, member 37 (Slc25a37) | 1.21 | 0.03 | 1.43 | <0.001 |
| NM_144793 | Solute carrier family 25, member 37 (Slc25a38) | -1.15 | 0.02 | -1.2 | 0.004 |

| **Gene Bank Acc** | **Gene** | **SFN2/ctrl** | | **SFN5/ctrl** | |
| --- | --- | --- | --- | --- | --- |
|  |  | **Abs FC** | **Fdr_P** | **Abs FC** | **Fdr_P** |
| NM_133670 | Sulfotransferase family 1a, phenol-preferring, member 1 (Sult1a1) | -1.005 | 0.97 | -1.326 | 0.029 |
| NM_016771 | Sulfotransferase family 1d, member 1 (Sult1d1) | 1.24 | 0.0357 | 1.49 | 0.046 |
| NM_139297 | UDP-glucose pyrophosphorylase 2 (Ugp2) | 1.19 | 0.001 | 1.2 | 0.0006 |
| NM_201641 | UDP glycosyltransferase 1 family, polypeptide a10 (Ugt1a10) | 1.13 | 0.073 | 1.73 | 0.028 |
| NM_201643 | UDP glucuronosyltransferase 1 family, polypeptide a5 (Ugt1a5) | 1.29 | 0.0163 | 1.545 | 0.012 |
| NM_201410 | UDP glucuronosyltransferase 1 family, polypeptide a6b (Ugt1a6b) | 1.81 | <0.0001 | 2.26 | <0.0001 |
| NM_201642 | UDP glucuronosyltransferase 1 family, polypeptide a7c (Ugt1a7c) | 1.51 | 0.096 | 1.66 | 0.026 |
| NM_201644 | UDP glucuronosyltransferase 1 family, polypeptide a9 | 1.31 | 0.0366 | 1.82 | 0.023 |

**Supplementary-Table 4:** Other SFN-modulated phase 2 enzymes
